# Individual Variability of CD19+ B‐Cell Repopulation in People With Multiple Sclerosis Treated With Extended Interval Dosing of Ocrelizumab

**DOI:** 10.1111/ene.70695

**Published:** 2026-07-07

**Authors:** Laura Hogenboom, Lisa G. Schoof, Liza M. Y. Gelissen, Jop Mostert, Luuk van Rooij, Elske Hoitsma, Caspar E. P. van Munster, Jeroen van Eijk, Nienke F. Kalkers, Kirsten S. Adriani, Anke Vennegoor, Ilse M. P. Arts, Liselore Mensing, Marijke Eurelings, Koen de Gans, Oliver Gerlach, Erwin L. J. Hoogervorst, Mark E. Kloosterziel, Jolijn J. Kragt, Ruben P. Portier, Ide Smets, Björn M. van Geel, Jessica Nielsen, Christiaan M. Roosendaal, Jurgen R. Piet, Esther Zeinstra, Onno N. Groeneveld, Bob W. van Oosten, Brigit A. de Jong, Bernard M. J. Uitdehaag, Eva M. M. Strijbis, Birgit I. Lissenberg‐Witte, Joep Killestein, Zoé L. E. van Kempen

**Affiliations:** ^1^ MS Center Amsterdam, Neurology, Vrije Universiteit Amsterdam, Amsterdam Neuroscience Amsterdam UMC Location VUmc Amsterdam the Netherlands; ^2^ Department of Neurology Rijnstate Ziekenhuis Arnhem the Netherlands; ^3^ Department of Neurology Maasstad Ziekenhuis Rotterdam the Netherlands; ^4^ Department of Neurology Alrijne Ziekenhuis Leiden the Netherlands; ^5^ Department of Neurology Amphia Ziekenhuis Breda the Netherlands; ^6^ Department of Neurology Jeroen Bosch Ziekenhuis 's Hertogenbosch the Netherlands; ^7^ Department of Neurology OLVG Amsterdam the Netherlands; ^8^ Department of Neurology Flevoziekenhuis Almere the Netherlands; ^9^ Department of Neurology Streekziekenhuis Koningin Beatrix Winterswijk the Netherlands; ^10^ Department of Neurology Spaarne Gasthuis Haarlem the Netherlands; ^11^ Department of Neurology Groene Hart Ziekenhuis Gouda the Netherlands; ^12^ Department of Neurology, Academic MS Center Zuyd Zuyderland Medical Center Sittard‐Geleen the Netherlands; ^13^ School for Mental Health and Neuroscience, Department of Neurology Maastricht University Medical Center Maastricht the Netherlands; ^14^ Department of Neurology Sint Antonius Ziekenhuis Utrecht the Netherlands; ^15^ Department of Neurology Wilhelmina Ziekenhuis Assen Assen the Netherlands; ^16^ Department of Neurology Reinier de Graaf Gasthuis Delft the Netherlands; ^17^ Department of Neurology Medisch Spectrum Twente Enschede the Netherlands; ^18^ Department of Neurology, MS Center ErasMS Erasmus Medisch Centrum Rotterdam the Netherlands; ^19^ Department of Neurology Noord West Ziekenhuisgroep Alkmaar the Netherlands; ^20^ Department of Neurology Ommelander Ziekenhuis Groningen the Netherlands; ^21^ Department of Neurology Slingeland Ziekenhuis Doetinchem the Netherlands; ^22^ Department of Neurology HagaZiekenhuis den Haag the Netherlands; ^23^ Department of Neurology Isala Klinieken Zwolle the Netherlands; ^24^ Department of Epidemiology and Data Science Vrije Universiteit Amsterdam Amsterdam the Netherlands; ^25^ Julius Center for Health Sciences and Primary Care, University Medical Center Utrecht Utrecht University Utrecht the Netherlands

**Keywords:** CD19+ B‐cells, intraindividual variability, ocrelizumab, personalised treatment, repopulation

## Abstract

**Background and Objectives:**

B‐cell repopulation patterns in ocrelizumab treated patients with multiple sclerosis are highly variable between individuals, but the course of B‐cell reoccurrence after subsequent doses within an individual is not yet determined. Our aim was to determine the intraindividual variability of CD19+ B‐cell repopulation after each ocrelizumab dose when using CD19+ B‐cell guided interval dosing.

**Methods:**

This was a prospective cohort study, as part of the ongoing BLOOMS trial, investigating participants randomised for B‐cell guided interval dosing of ocrelizumab with ≥ 2 dosing intervals. Coefficients of variation were calculated for time from last ocrelizumab dose to first CD19+ B‐cell measurement ≥ 0.01 × 10^9^ cells/L.

**Results:**

Seventy‐five participants with a total of 209 B‐cell guided intervals were included. Time from last dose to first appearance of CD19+ B‐cell count ≥ 0.01 × 10^9^ cells/L showed wide variability between individuals (20.6–72.1 weeks), but the median variation was 5.6% (IQR: 3.1–8.4) within an individual. This translated to a variation of 2 weeks per dosing interval on average.

**Discussion:**

Time to CD19+ B‐cell repopulation after each ocrelizumab dose is individually stable. This finding could pave the way for easier and more accessible future personalised interval dosing of B‐cell depleting therapies if this stability is confirmed in long‐term treatment.

## 1 Introduction

B‐cell depleting therapies are highly effective in suppressing inflammatory disease activity in people with multiple sclerosis (MS), but are also associated with side effects, such as infections, hypogammaglobulinemia and a poor humoral vaccination response [1, 2, 3, 4, 5, 6]. Additionally, treatment convenience has become a key consideration given the impact of therapy on patients' quality of life and daily functioning [7]. Optimization of dosing strategies could integrate considerations of efficacy, safety, patient‐centred outcomes and reduce health care costs [8]. Since anti‐CD20 therapies cause long‐term sustained B‐cell depletion, B‐cell guided dosing could be a promising alternative to standard dosing [9]. Our aim was to determine intraindividual stability of time to B‐cell repopulation after an ocrelizumab dose when using CD19+ B‐cell guided dosing.

## 2 Methods

This was a prospective cohort study, as part of a subgroup analysis of the ongoing randomised controlled trial BLOOMS (ClinicalTrials.gov Identifier: NCT05296161). The BLOOMS trial compares two dosing strategies of intravenous ocrelizumab (1:1): (I) standard dosing of 600 mg every 6 months and (II) CD19+ B‐cell tailored interval dosing in which 600 mg is administered after the CD19+ B‐cell exceeds the redosing threshold of ≥ 0.01 × 10^9^ cells/L. Participants that were randomised for B‐cell tailored dosing with at least two dosing intervals within the follow‐up of the study were included (as of August 2025). Patient characteristics, disease history and clinical information were collected at the baseline study visit, which was planned 6 months after the last ocrelizumab dose.

The BLOOMS study protocol was approved by the medical ethics committee (VUMC Ethics committee 2021.0639). Participants were included in 22 Dutch hospitals and all participants signed a written informed consent.

### 2.1 BLOOMS Trial Protocol

Inclusion criteria for the BLOOMS trial are a diagnosis of relapsing onset MS according to McDonald 2017 criteria [10], two full ocrelizumab dosing cycles, clinically stable disease for at least 3 months prior to inclusion and no prior treatment with alemtuzumab, cladribine or stem cell transplantation. In the personalised dosing group, CD19+ B‐cells in serum were measured monthly, starting 24 weeks after last ocrelizumab dose, and only stopped when the CD19+ B‐cells exceeded ≥ 0.01 × 10^9^ cells/L. Per protocol, an ocrelizumab infusion was scheduled within 4 weeks after exceeding this re‐dosing threshold (Figure S1).

### 2.2 Outcomes Measures

The time interval between last ocrelizumab infusion and first CD19+ B‐cell repopulation ≥ 0.01 × 10^9^ cells/L was analysed in weeks. To take into account the influence of artificially stable 6‐month dosing intervals for participants that could not extend their ocrelizumab infusion due to CD19+ B‐cell repopulation ≥ 0.01 × 10^9^ cells/L at the first CD19+ B‐cell measurement at 24 weeks after last dose, the outcome was analysed for both (I) all participants randomised for B‐cell guided dosing, and (II) for participants that had at least one extended interval ≥ 28 weeks during follow‐up. Intra‐individual variability for the interval between last infusion and first occurrence of CD19+ B‐cell count ≥ 0.01 × 10^9^ cells/L was quantified using the coefficient of variation (CV). The CV was calculated for each unique participant by dividing the standard deviation of their individual interval durations by their individual mean duration, expressed as a percentage. Sensitivity analyses were performed for all participants with ≥ 3 B‐cell guided dosing intervals.

### 2.3 Statistical Analyses

Statistical analyses were performed using R studio 4.3.2. Descriptive data were presented as means with standard deviation (SD) for normally distributed data, medians with interquartile range (IQR) for non‐normally distributed data, or frequencies with percentages for categorical data. Coefficients of variation were calculated based on all B‐cell tailored dosing intervals of each individual.

## 3 Results

Seventy‐five participants with a total of 209 B‐cell tailored intervals were included (Figure S2). All participants had ≥ 2 B‐cell tailored dosing intervals, as this was an inclusion criterium; however, 49.3% (*n* = 37) had ≥ 3 dosing intervals during follow‐up. Further baseline characteristics can be found in Table 1.

**TABLE 1:** Patient characteristics.

Total cohort, *n*	75
Age, mean (SD)	40.0 (8.8)
Female, *n* (%)	49 (65.3%)
BMI in kg/m^2^, median (IQR)	24.5 (22.6–26.4)
Disease duration in years, mean (SD)	9.8 (6.7)
EDSS at baseline, median (IQR)	2.0 (1.5–3.0)
Number of ocrelizumab infusions before baseline, mean (SD)	5.1 (2.6)
Baseline CD19+ B‐cell count in cells/μL, median (IQR)	2.0 (1.0–10.0)
Number of B‐cell guided dosing intervals in BLOOMS, *n* (%)	
Two intervals	38 (50.6%)
Three intervals	19 (25.3%)
Four intervals	15 (20.0%)
≥ 5 intervals	3 (4%)

*Note:* Baseline characteristics were established at the baseline visit of the BLOOMS trial, always scheduled 20–24 weeks from last ocrelizumab dose. Mean values are displayed with ± standard deviation. Median values are displayed with (interquartile range). Frequencies are displayed with (percentages).

Abbreviation: EDSS, expanded disability status scale.

### 3.1 Inter‐Individual Variability of Intervals

**FIGURE 1:**
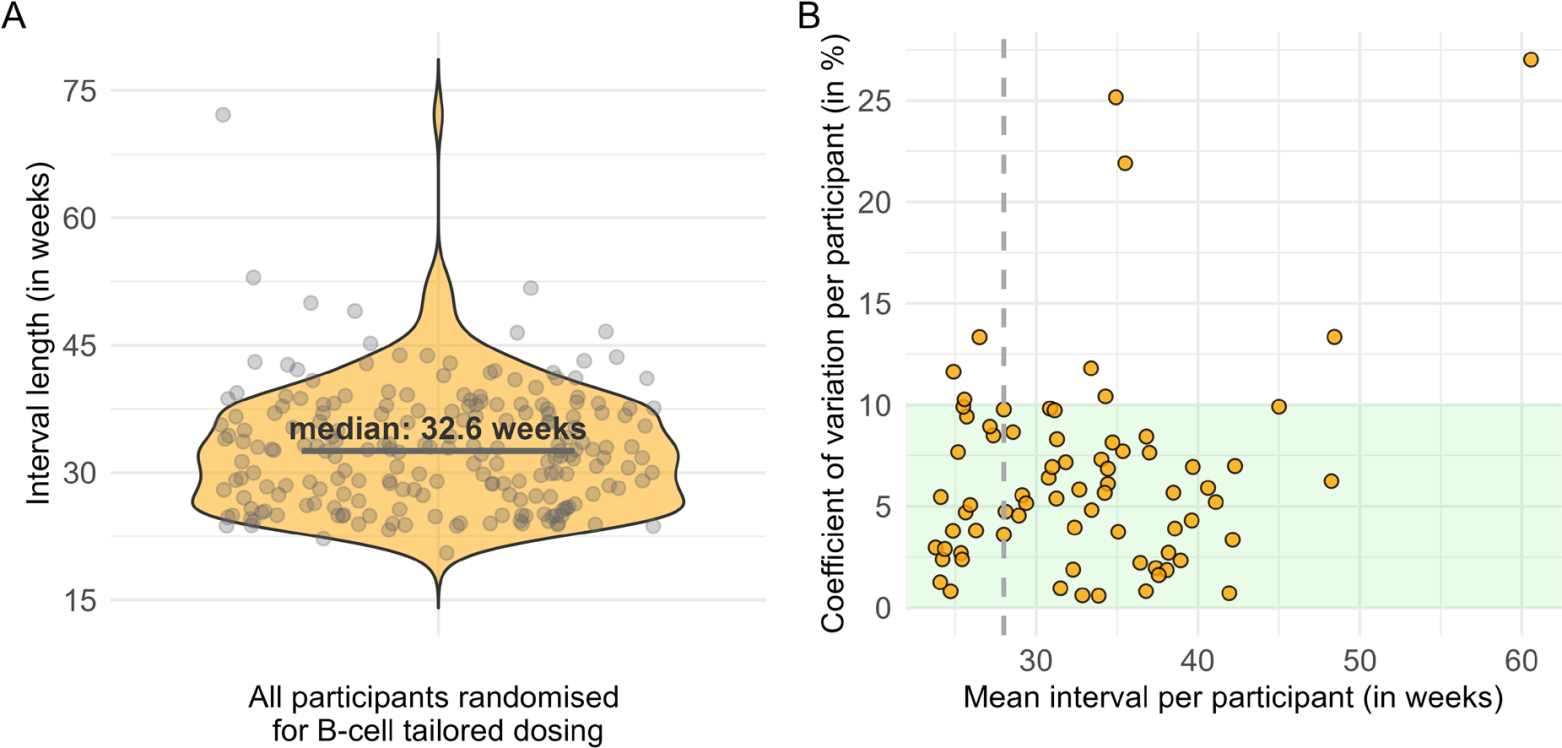
(A) Interval length of ocrelizumab infusion to first CD19+ B‐cell count exceeding the re‐dosing threshold of ≥ 0.01 × 10^9^ cells/L of all participants randomised for B‐cell tailored dosing, presented with the median in weeks. (B) Coefficients of variation per participant in percentage, displayed by average interval duration per participant. The green area represents ≤ 10% of variation per individual. The vertical grey line represents a standard, not extended interval of ≤ 28 weeks.

The time between last ocrelizumab dose to first CD19+ B‐cell count ≥ 0.01 × 10^9^ cells/L ranged from 20.6 to 72.1 weeks (mean: 32.8 ± 7.0) (Figure 1A and Table 2). The majority of participants (80%) received extended interval dosing ≥ 28 weeks at least once, while 20% of participants had CD19+ B‐cell repopulation above the re‐dosing threshold before 28 weeks after last ocrelizumab dose.

**TABLE 2:** Description of individual interval variation in all subgroups.

	Infusion to first CD19+ B cell ≥ 0.01 × 10^9^ cells/L	
	All patients (*n* = 75)	Patients with at least one interval ≥ 28 weeks (*n* = 60)
Interval in weeks, mean ± SD	32.9 ± 6.9	35.0 ± 6.2
Individual coefficient of variation in %, median (IQR)	5.6 (3.1–8.4)	5.7 (3.4–7.7)
Percentage that has ≤ 10% variation in B‐cell tailored dosing intervals, in %	87.8	88.0

*Note:* Participants were only included in the subgroup with extended dosing if at least one CD19+ B‐cell tailored dosing interval was ≥ 28 weeks.

### 3.2 Intra‐Individual Interval Stability

The median of all individual coefficients of variation was 5.6% (IQR: 3.1–8.4) (Table 2). On average, this translates to a variation of 1.8 weeks per individual dosing interval, when considering the mean extended dosing interval of 32.8 weeks. Less than 10% variability was observed in the vast majority of patients (87.8%) (Table 2 and Figure 1B). Similar results were found in those with at least one interval ≥ 28 weeks, hereby excluding the participants with B‐cell repopulation above the re‐dosing threshold at the first CD19+ B‐cell measurement. Sensitivity analyses of a subgroup with ≥ 3 B‐cell tailored dosing intervals (*n* = 37 and *n* = 30) confirmed these findings (Table S1).

### 3.3 Relapse Incidence

Only one clinical relapse was reported in this subgroup of the BLOOMS trial during follow‐up, occurring in a participant with a mean dosing interval of 28 weeks (CV = 9.8%).

## 4 Discussion

Our findings show that while time to B‐cell repopulation after an ocrelizumab infusion varies greatly between individuals, this interval remains relatively stable over time within each individual. On average, less than 6% variation per individual was found in time to first CD19+ B‐cell repopulation ≥ 0.01 × 10^9^ cells/L, which translates to less than 2 weeks variability per dosing interval in an average extended dosing interval of 32 weeks.

Several temporal predictors for B‐cell dynamics during ocrelizumab are previously identified, of which age and cumulative dose are the most profound, and could therefore influence the stability of the individual dosing interval during long‐term treatment [11, 12, 13]. We recently reported a decrease in CD19+ B‐cell count during ocrelizumab treatment of 1.3% and 7.0% for respectively each additional year of age and each cumulative dose, in a larger (*n* = 567) retrospective cohort. Similarly, Disanto et al. reported that with each additional year of age there is an 18% lower risk of having CD19+ B‐cells ≥ 0.001 × 10^9^ cells/L within a normal dosing regimen [11]. However, in the context of significant change of an individual CD19+ B‐cell guided interval, it would take several years to translate the effect of additional years of age and cumulative doses to a clinically significant difference in interval length of more than 1 month extension. Therefore, although the effect of these variables should not be underestimated, they are primarily relevant in a long‐term context. Subsequently, these variables, along with patient‐specific characteristics such as weight and prior therapy use, cannot fully explain the wide interindividual differences in time to B‐cell repopulation after an ocrelizumab infusion. It is therefore helpful for B‐cell guided dosing to be able to lean on these findings of relative stability of individual intervals.

Our study has some limitations, including the relatively short follow‐up duration of a maximum of five B‐cell tailored dosing intervals and the small cohort of 75 participants. However, sensitivity analysis in participants with ≥ 3 B‐cell tailored dosing intervals revealed similar results as the primary analysis (CV: 5.9% vs. 5.6%), thereby supporting the validity of the primary results. Although the re‐dosing threshold of ≥ 0.01 × 10^9^ cells/L can be considered arbitrary, it is nonetheless in line with what is clinically considered as a sign of B‐cell repopulation in literature and is grounded in experience during the COVID‐pandemic [14, 15, 16]. In addition to these considerations of the best re‐dosing threshold, the question persists as to whether CD19+ B‐cell dynamics are the ideal biomarker to monitor the efficacy of ocrelizumab. Despite the known role that CD27+ memory B‐cells play in the pathology of MS [17], the benefits of tracking this specific subset are not yet established. Among patients dosed based on a CD27+ guided dosing approach, disease activity is generally low, making it difficult to establish a clear threshold for breakthrough disease [11, 18].

Whether or not CD19+ B‐cells represent an ideal biomarker, these findings carry significant clinical implications when clinicians implement an extended dosing strategy in patients with low IgG levels or frequent infections during ocrelizumab treatment. If the BLOOMS trial eventually proves non‐inferiority of CD19+ B‐cell guided dosing compared to standard 6‐monthly dosing, these findings on stability of intra‐individual dosing intervals will significantly impact the cost‐effectiveness of this costly therapy and patient convenience.

## 5 Conclusion

This preliminary subgroup analysis of the BLOOMS trial shows a wide range of time to CD19+ B‐cell repopulation across patients treated with ocrelizumab, but minimal variability in time to CD19+ B‐cell repopulation after each ocrelizumab dose within individuals. This is a promising finding, paving the way for easier and more accessible future implementation of personalised treatment in MS.

## Author Contributions

**Luuk van Rooij:** writing – review and editing, investigation. **Caspar E. P. van Munster:** writing – review and editing, investigation. **Elske Hoitsma:** conceptualization, writing – review and editing, investigation. **Nienke F. Kalkers:** writing – review and editing, investigation. **Liza M. Y. Gelissen:** writing – review and editing, investigation. **Kirsten S. Adriani:** writing – review and editing, investigation. **Marijke Eurelings:** writing – review and editing, investigation. **Ilse M. P. Arts:** writing – review and editing, investigation. **Ruben P. Portier:** writing – review and editing, investigation. **Anke Vennegoor:** writing – review and editing, investigation. **Liselore Mensing:** writing – review and editing, investigation. **Onno N. Groeneveld:** writing – review and editing, investigation. **Jolijn J. Kragt:** writing – review and editing, investigation. **Koen de Gans:** writing – review and editing, investigation. **Mark E. Kloosterziel:** writing – review and editing, investigation. **Ide Smets:** writing – review and editing, investigation. **Laura Hogenboom:** conceptualization, writing – review and editing, writing – original draft, visualization, formal analysis, project administration, data curation, methodology, investigation. **Björn M. van Geel:** writing – review and editing, investigation. **Esther Zeinstra:** writing – review and editing, investigation. **Jessica Nielsen:** writing – review and editing, investigation. **Bernard M. J. Uitdehaag:** conceptualization, writing – review and editing, investigation. **Oliver Gerlach:** writing – review and editing, investigation. **Christiaan M. Roosendaal:** writing – review and editing, investigation. **Jurgen R. Piet:** writing – review and editing, investigation. **Jop Mostert:** conceptualization, writing – review and editing, investigation. **Zoé L. E. van Kempen:** conceptualization, writing – review and editing, writing – original draft, supervision, visualization, project administration, funding acquisition, data curation, investigation. **Birgit I. Lissenberg‐Witte:** methodology, writing – review and editing, formal analysis. **Lisa G. Schoof:** writing – review and editing, investigation. **Jeroen van Eijk:** writing – review and editing, investigation. **Bob W. van Oosten:** conceptualization, writing – review and editing, investigation. **Brigit A. de Jong:** conceptualization, writing – review and editing, investigation. **Eva M. M. Strijbis:** conceptualization, writing – review and editing, investigation. **Joep Killestein:** conceptualization, writing – review and editing, supervision, funding acquisition, investigation. **Erwin L. J. Hoogervorst:** conceptualization, writing – review and editing, investigation.

## Funding

This research was funded by ZonMw (No. 848044001) and TreatMeds.

## Ethics Statement

The BLOOMS study protocol was approved by the medical ethics committee (VUMC Ethics committee 2021.0639).

## Consent

All participants signed a written informed consent.

## Conflicts of Interest

L.H., L.G.S., L.M.Y.G., J.M., L.R., C.E.P.M., N.F.K., K.S.A., A.V., I.M.P.A., L.M., M.E., O.G., E.L.J.H., M.E.K., J.J.K., R.P.P., B.M.G., J.N., J.R.P., E.Z., O.N.G., B.W.O., B.A.J., E.M.M.S., B.I.L.‐W. and Z.L.E.K. have nothing to report. E.H. received speaker and congress fees from Merck Serono, Biogen Idec, Roche, and Sanofi Genzyme. J.E. received consultancy fees and/or speaker honoraria from Roche, Biogen, Teva, Merck, Novartis and Sanofi/Genzyme. K.G. served on scientific advisory boards for Roche, Janssen, Sanofi‐Genzyme, Novartis and Merck, received conference fee and travel support from Novartis, Biogen, Sanofi‐Genzyme, Teva, Abbvie and Merck and received educational event support from Novartis. I.S. received speakers' honoraria from Merck, Biogen Idec and Sanofi. C.M.R. received a congress fee from Novartis. B.M.J.U. received consultancy fees from Immunic Therapeutics (adjudication committee; payments to institution). J.K. received consulting fees for F. Hoffmann‐La Roche, Biogen, Teva, Merck, Novartis and Sanofi/Genzyme (payments to institution); reports speaker relationships with F. Hoffmann‐La Roche, Biogen, Teva, Merck, Novartis and Sanofi/Genzyme (payments to institution); adjudication committee of MS clinical trials of Immunic (payments to institution).

## Supporting information


**Figure S1:** CD19+ B cell measurements in BLOOMS trial (ClinicalTrials.gov Identifier: NCT05296161). After randomisation for personalised dosing, CD19+ B‐cells are measured starting 24 weeks after last ocrelizumab infusion as intervals are never shortened. If the B‐cell count is < 0.01 × 10^9^ cells/L, the measurement is repeated 4 weeks later, until the CD19+ B‐cells exceed this re‐dosing threshold.
**Figure S2:** Patient disposition.
**Table S1:** Sensitivity analyses on participants with at least 3 B‐cell tailored dosing intervals.

## Data Availability

Deidentified data will be shared upon reasonable request from any qualified investigator.
