# Supplementary material for: Individual Variability of CD19+ B‐Cell Repopulation in People With Multiple Sclerosis Treated With Extended Interval Dosing of Ocrelizumab
Source: Eur J Neurol. 2026 Jul 7;33(7):e70695. doi: 10.1111/ene.70695 (PMC13339935; doi:10.1111/ene.70695)
Supplement: Supplementary file 1 — Figure S1: CD19+ B cell measurements in BLOOMS trial (ClinicalTrials.gov Identifier: NCT05296161). After randomisation for personalised dosing, CD19+ B‐cells are measured starting 24 weeks after last ocrelizumab infusion as intervals are never shortened. If the B‐cell count is < 0.01 × 10^9^ cells/L, the measurement is repeated 4 weeks later, until the CD19+ B‐cells exceed this re‐dosing threshold. Figure S2: Patient disposition. Table S1: Sensitivity analyses on participants with at least 3 B‐cell tailored dosing intervals. [file ENE-33-e70695-s001.docx]

**Supplemental material**

**
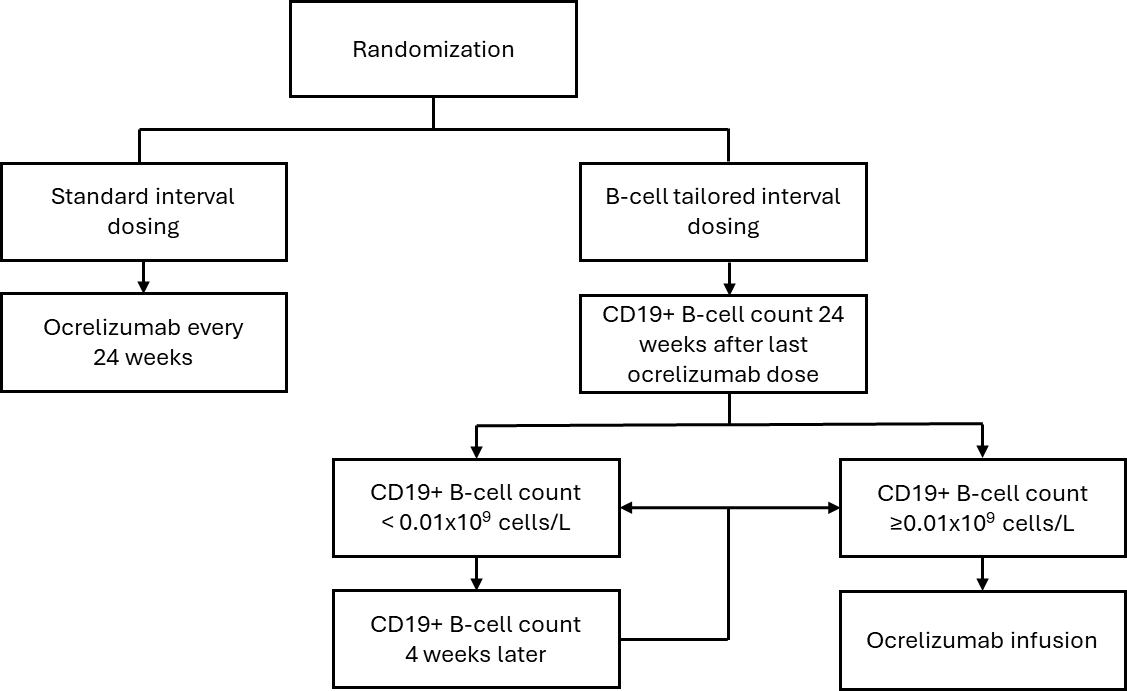
**

**Supplementary figure 1.** CD19+ B cell measurements in BLOOMS trial (ClinicalTrials.gov Identifier: NCT05296161). After randomization for personalised dosing, CD19+ B-cells are measured starting 24 weeks after last ocrelizumab infusion as intervals are never shortened. If the B-cell count is <0.01x10^9^ cells/L, the measurement is repeated four weeks later, until the CD19+ B-cells exceed this re-dosing threshold.


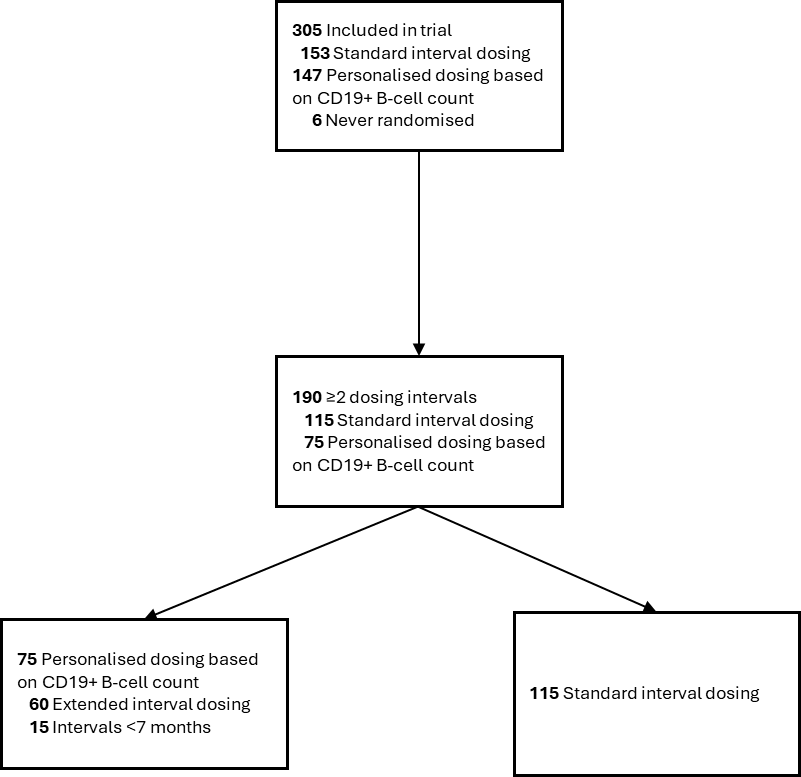


**Supplementary figure 2**. Patient disposition

**Supplementary table 1.** Sensitivity analyses on participants with at least 3 B-cell tailored dosing intervals.

|  |  | **Infusion to first CD19+ B cell ≥10 cells/µL interval** | |
| --- | --- | --- | --- |
|  |  | **All patients (*n*=37)** | **Patients with at least one interval ≥28 weeks (*n*=30)** |
| Interval in weeks, mean ±SD, |  | 33.3 ±6.2 | 35.2 ±5.2 |
| Individual coefficient of variation in %, median (IQR), |  | 5.9 (4.7-7.3) | 6.2 (4.9-7.2) |
| Percentage that has ≤10% variation in B-cell tailored dosing intervals, in % |  | 91.9 | 93.3 |
